# Don't be blue: Pure green light spurs brassinosteroid signaling

**DOI:** 10.1093/plcell/koad040

**Published:** 2023-02-18

**Authors:** Nora Flynn

**Affiliations:** The Plant Cell, American Society of Plant Biologists, USA; Department of Botany and Plant Sciences, University of California Riverside, Riverside, CA, USA

Plants grow toward the light, but not all wavelengths of light are equally desirable. Red and blue wavelengths are readily absorbed by leaves because they are highly effective for photosynthesis. On the other hand, green light is mostly reflected and transmitted, giving leaves their familiar green color. Due to this biased absorbance, desirable wavelengths become sparse for seedlings under a canopy, leaving them with a larger percentage of green light. What do seedlings do with this green light?

Fortunately for shaded plants, the role of light extends beyond photosynthesis. Light is also a critical regulator of plant development and plants can respond to different wavelengths uniquely. For example, red and blue wavelengths are sensed by photoreceptors that strongly promote photomorphogenesis (Wang et al. 2018; Yoo et al. 2019). Without sufficient red or blue light, a seedling instead focuses on hypocotyl elongation to extend toward any available light.

Unlike red and blue light, green light's effects on plants are more obscure. Studies waver on green light's developmental role and mode of perception. The conflicting results are due to contaminating wavelengths found in many commercial green light sources, entangling green light response with responses to other wavelengths. In this issue of *The Plant Cell*, **Yuhan Hao and colleagues** (Hao et al. 2023) report that “pure” green light does indeed promote hypocotyl elongation through a process triggered by brassinosteroid signaling.

To clarify the relationship between the green light and hypocotyl elongation, the authors grew Arabidopsis in darkness, commercial-grade green light, or well-filtered green light, where a filter removed any contaminating blue light (<500 nm). When grown in the filtered green light, seedlings had even longer hypocotyls than seedlings grown in the dark. Meanwhile, seedlings grown in unfiltered green light had short hypocotyls due to blue light contamination. Therefore, even low levels of blue light prompt photomorphogenesis, but pure green light stimulates hypocotyl elongation for shade avoidance.

How does green light exposure result in hypocotyl elongation? After growing in darkness or filtered green light, a variety of mutants deficient in photosynthesis or known photoreceptors all retained the wild-type response of longer hypocotyls in green light than in darkness. Seemingly, the green light response is therefore not dependent on photosynthesis or on the tested photoreceptors.

**Figure 1.**
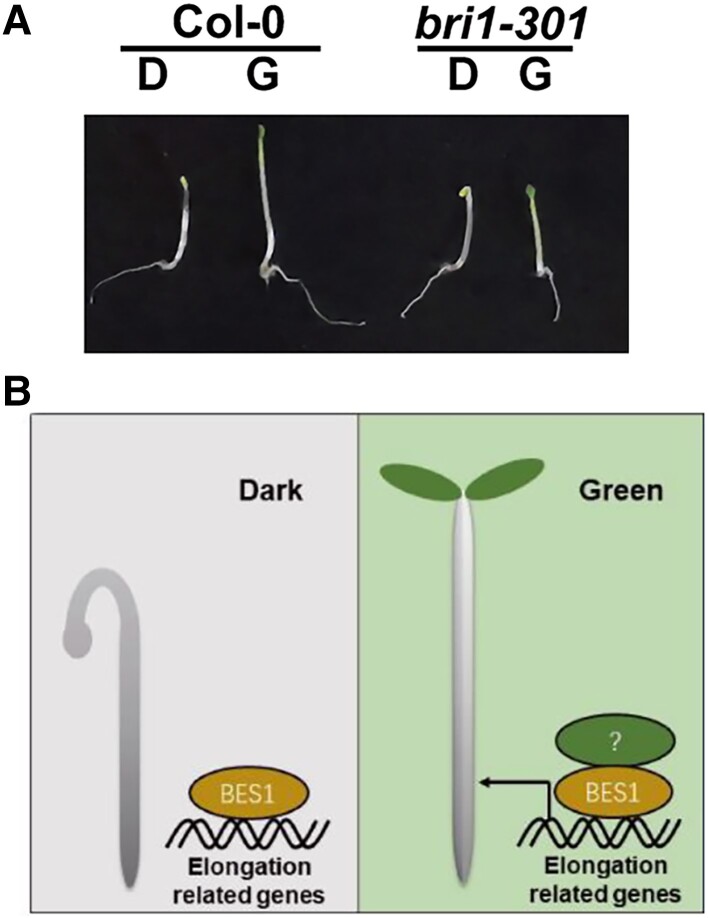
Green light activates BR signaling. A) Wild-type (Col-0) and BR receptor-deficient (*bri1-301*) seedlings grown in darkness (D) or green light (G). B) Green light promotes hypocotyl elongation through BES1 DNA binding; a potential green light receptor interacts with BES1 to enhance its DNA binding activity. Adapted from Hao et al. (2023), Supplemental Figs. 4A and S5D.

To identify new candidates involved in the green light response, the authors analyzed RNA-seq data for seedlings grown entirely in the dark or transferred to filtered green light. About 40% of the differentially expressed genes between these 2 groups were regulated by brassinosteroids (BR), plant hormones involved in hypocotyl elongation. To examine if the BR signaling pathway is required for hypocotyl elongation in the green light, mutants deficient in BR biosynthesis and perception were grown in darkness or filtered green light. These mutants were insensitive to green light and did not show longer hypocotyls than in the dark (Fig. 1A), suggesting that BRs are necessary for the green light response.

If the BR signaling pathway is involved in the green light response, the authors reasoned that transcription factors in this pathway should demonstrate altered DNA binding under green light conditions. Using ChIP-qPCR, the authors examined the DNA binding of BRI1-EMS-SUPPRESSOR 1 (BES1), a master transcription factor in the BR signaling pathway (Yang et al. 2011). In green light, BES1 showed increased binding to expected promoters, indicating that green light positively affects BES1 DNA binding to regulate transcription and drive hypocotyl elongation (Fig. 1B).

This study demonstrates that the BR signaling pathway is required to promote hypocotyl growth under green light. Although the green light receptor remains to be revealed, this study takes a promising step toward solving the puzzle of shade signaling in green light.
